# The Impact of a Communitywide Smoke-Free Ordinance on Smoking Among Older Adults

**Published:** 2008-12-15

**Authors:** John D. Prochaska, James N. Burdine, Marcia G. Ory, Joseph R. Sharkey, Kenneth R. McLeroy, Brian Colwell, Nelda Mier, Kendra Bigsby

**Affiliations:** Texas A&M Health Science Center School of Rural Public Health; Texas A&M Health Science Center School of Rural Public Health, College Station, Texas; Texas A&M Health Science Center School of Rural Public Health, College Station, Texas; Texas A&M Health Science Center School of Rural Public Health, College Station, Texas; Texas A&M Health Science Center School of Rural Public Health, College Station, Texas; Texas A&M Health Science Center School of Rural Public Health, College Station, Texas; Texas A&M Health Science Center School of Rural Public Health, College Station, Texas; Health District of Northern Larimer County, Fort Collins, Colorado

## Abstract

**Introduction:**

Clean-air and smoke-free ordinances have been shown to reduce the prevalence of smoking among the overall population, but their effects on the smoking prevalence among older adults deserves further attention. We examined changes in self-reported cigarette smoking and in attitudes toward smoking after the implementation of such ordinances in Fort Collins, Colorado, in 2003.

**Methods:**

Communitywide health status surveys were mailed out to northern Larimer County residents recruited via random-digit dialing in 2001 and 2004. Secondary data analysis was conducted for respondents living in Fort Collins, comparing the entire sample with a subsample of adults aged 50 years or older. Univariate analyses were used to determine differences in self-reported cigarette smoking between the groups across the 2 surveys. Multivariate logistic regression models estimated differences in smoking status and in attitudes toward acceptability of public smoking between the 2 survey administrations, controlling for demographic correlates.

**Results:**

Smoking rates among older respondents failed to change, despite significant decreases in smoking rates in the entire adult population. Furthermore, attitudes toward smoking in public did not change between the 2 surveys for either of the groups.

**Conclusion:**

Different factors may influence the decision to stop smoking for older adults and younger adults. We recommend the use of multiple approaches on different ecological levels to ensure that communitywide antismoking intervention efforts reach all population segments.

## Introduction

Smoking, a leading cause of preventable death in the United States, is associated with increased risk of developing a host of chronic diseases (including cardiovascular disease, pulmonary disease, and several forms of cancer) and is linked with decreased life expectancy and quality of life (1,2). Smoking rates are declining in the United States, and 2002 marked the first time that more than half of US residents who had ever smoked had quit (3). Researchers have directed considerable attention to increasing smoking cessation rates in the broader population, but more information about older smokers is needed (4).

Smoking rates are lower in adults aged 50 years or older than in younger adults; nevertheless, nearly 22% of older adults are smokers (5). Older smokers are at greater risk than younger smokers for developing a range of chronic illnesses and dying prematurely because they have smoked longer (averaging 40 years of smoking), tend to be heavier smokers, and are more likely to develop smoking-related illnesses (6). They are also less likely to believe that smoking harms their health (6). Older smokers can benefit from smoking cessation by lowering their risk of premature death and their risk of developing coronary disease, chronic obstructive pulmonary disease, and some forms of cancer. Furthermore, older smokers who cease using tobacco have higher levels of physical function and better quality of life (7).

Public health policy interventions aimed at organizational and community levels (such as increased taxation of tobacco products and workplace smoking ordinances) cost-effectively reduce smoking prevalence in broad populations by limiting purchase and by reinforcing negative social norms for smoking (8-12). Implementation of such policies has little to no negative effect on the local economy, reaches a broad target population, and is associated with decreasing rates of primary smoking (9,13-18).

Studies on such policies have examined the impact of communitywide antismoking policies on smokers in general, but little research has addressed their effect on older smokers in particular. Initial results suggest that such policies may aid older smokers in reducing or quitting smoking (19); however, research is needed to validate these findings.

Data collected before and after the passage of antismoking ordinances may provide insight into their effectiveness among older adults relative to other age groups. Such an opportunity occurred in 2003 when the citizens of Fort Collins, Colorado (estimated population of 126,967 in 2004) (20), passed a smoke-free work and public place ordinance restricting the right to smoke in a range of public places, aimed at reducing public exposure to secondhand smoke. This ordinance restricts smoking in restaurants, bars, places of employment, and within 20 feet of an entrance to such locations. However, smoking is permitted in private residences, up to 25% percent of rooms in a hotel or motel, tobacco stores, and other locations. Researchers were able to use the policy changes as a natural experiment to retrospectively study the effects of tobacco ordinances on older smokers. This experiment was accomplished through a community-based participatory approach whereby academic researchers at the Texas A&M Health Science Center worked closely with local researchers and their local community health service organization partners in Fort Collins to identify possible survey questions and research methods and assist with data interpretation.

In 2001 and 2004, the Health District of Northern Larimer County, with input from community and academic partners, conducted community health surveys to collect cross-sectional data on the health and health-related behaviors (including current cigarette use) of the population served by the health district, an area that encompasses the northern two-thirds of Larimer County, Colorado, and includes the city of Fort Collins. This study examines self-reported smoking and attitudes toward smoking in public places before and after the implementation of a communitywide smoking ordinance among adults aged 50 years or older, relative to other age groups.

The major objectives of this research were 1) to estimate changes in smoking levels and attitudes toward public smoking before and after a communitywide smoking ban, 2) to examine factors associated with changes in smoking among respondents to the health survey, and 3) to examine the differences in smoking levels between survey administrations for the entire sample and for respondents aged 50 or older. Because the implementation of the ordinances should reduce the available areas for residents to smoke in public and potentially change the social norms in a community toward smoking in public, we anticipated a reduction in the number of smokers. Although few data were available, we anticipated that the effect on younger and older populations would differ. Although older adults are more likely to have used nicotine for a longer period of time (6), it was expected that older adults would be less influenced by the ordinances not because they had been smoking longer but because they did not frequent the facilities covered by the ordinances.

## Methods

### Sample design and recruitment strategies

The 2001 and 2004 community health surveys, conducted by the health district, were self-administered, mailled surveys distributed to 2 cross-sectional cohorts of people aged 18 or older living within the health district service area and within the rest of Larimer County. The 2 surveys were similar in design and content, with only minor changes made to each to address specific local concerns at time of survey collection. Data on the variables analyzed in this study were collected by using identical questions on both surveys. Both surveys used the same sampling and recruitment methods.

Potential participants were screened via random-digit–dialed telephone calls. Using a list of randomized telephone numbers with relevant area codes and prefixes for the study area, telephone operators working for a commercial survey research firm attempted to contact each number a maximum of 4 times. Protocols excluded telephone numbers to businesses, cell phones, and other nonresidence numbers. To reduce the potential for sex bias, the operator asked to speak with the person living in the household older than 18 years who had the next birthday. Detailed data about participants and refusals from the telephone screening stage were not collected.

Staff mailed an informational letter, survey instrument, postage-paid return envelope, and monetary consideration ($2) to respondents who agreed to participate in the study. A second survey packet was sent after 2 weeks as a reminder. Returned surveys were reviewed for completeness and eligibility for incorporation into the dataset.

For the 2001 survey, telephone operators successfully contacted 4,381 eligible people in Larimer County, and of the 3,125 (71.3%) who agreed to participate, 2,295 (73.4%) returned surveys (Figure). Of the 2,272 surveys deemed usable for inclusion in the dataset, 1,681 (74.0%) were from residents of Fort Collins, and the remainder were from residents outside of Fort Collins but within Larimer County.

**Figure 1:**
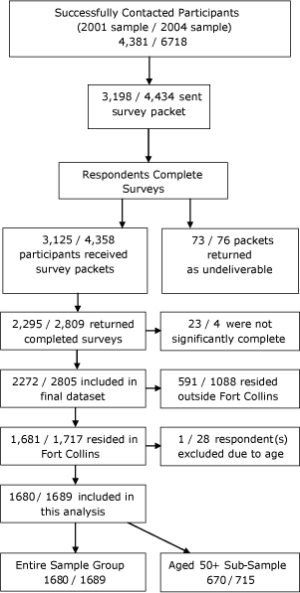
Recruitment flow chart for survey on current smoking status and attitudes toward smoking, Fort Collins, Colorado, 2001 and 2004.

For the 2004 survey, operators successfully contacted 6,718 people in Larimer County and of the 4,434 (66.0%) who agreed to participate, 2,809 (63.4%) returned surveys. Of the 2,805 surveys deemed usable for inclusion in the dataset, 1,717 (60.9%) were from residents of Fort Collins, and the remainder were from residents outside of Fort Collins but within Larimer County. One respondent from the 2001 survey and 28 respondents from the 2004 survey were excluded because of age. The final dataset consisted of 3,369 responses from adults aged 18 or older living in Fort Collins in 2001 combined with responses from adults aged 21 or older in 2004. The difference in age selection between the 2 surveys is to account for the aging of the cohort from which the samples are drawn. This does not control for migration in and out of the area, but it does help ensure that the samples included in the analyses were more likely drawn from a similar population cohort.

### Measures

The major variable of interest was cigarette smoking. In these surveys, cigarette smoking was measured as a dichotomous response to the question, "Do you currently smoke cigarettes?" and coded as yes or no. Attitude toward the social acceptability of public smoking was measured as a categorical variable by asking respondents to rate their agreement with the statement, "It is socially acceptable to smoke in public," on a 5-point Likert scale, with "strongly agree" coded 1. For analysis, considering that a change toward disagreement is desired, the variable was collapsed into a single dummy variable, with "strongly disagree" and "disagree" being coded as 1.

Age was recorded as a continuous variable in years. Older adults were considered aged 50 years or older. Sex was recorded as a dichotomous variable (female = 1). Income was collected as the total household income for the previous year in dollars and was then coded into a categorical variable with 3 categories: poverty (incomes at or below the federal poverty guidelines at year of survey), low-income (incomes 101% to 185% of the federal poverty at year of survey), and above low income (incomes above 185% of the federal poverty level at year of survey). Racial/ethnic status was collected as African American, white non-Hispanic, Hispanic/Latino, Asian/Pacific Islander, and Native American/Alaskan Native, then converted into white non-Hispanic versus other. Marital status was collected as married, widowed, divorced/separated, single never married, and living with partner, then converted to married versus unmarried.

### Analysis

Univariate descriptive statistics were calculated for the measures by survey year and by sample (overall sample and those aged 50 or older). A multivariate logistic regression model was used to estimate the change in odds of self-reported smoking between the 2 surveys, controlling for the demographic correlates. A second multivariate logistic regression model was used to estimate the change in odds of indicating social acceptance of public smoking between surveys, controlling for status as a smoker. All data were analyzed using Stata 9.2 (Stata Corp, LP, College Station, Texas), by robust White-Huber standard errors were computed.

Although the survey targeted residents from all of Larimer County, this analysis included data only for those living in Fort Collins, where the public smoking ordinance was implemented. Two analytical samples were selected from both survey administrations (2001 and 2004). The first included all respondents aged 18 or older in 2001 and aged 21 or older in 2004 (all-ages sample). Respondents for the older adult sample were defined as being aged 50 years or older at time of the 2001 survey and 53 or older at time of the 2004 survey.

The 2001 sample included 1,680 adults aged 18 or older, including 670 adults aged 50 or older. The 2004 sample included 1,689 adults aged 21 or older, of whom 715 adults were aged 53 or older. We estimated the degree to which the proportion of individuals currently smoking cigarettes changed significantly between the 2 survey years by using robust multiple logistic regression models that controlled for age, sex, marital status, race, and income. A measure of years of education is reported in the samples' summary statistics (Table 1) but was not included in the logistic regression models because of its collinear interaction with income and low variability within the sample. Finally, with the same demographic covariates, we used a model to determine the existence of significantly different odds of reporting smoking between the 2 survey periods for adults aged 50 to 59 and for adults aged 60 or older. This final analysis was conducted to determine whether there was any difference within the older adult subsample.

## Results

The demographic characteristics of the samples are summarized in Table 1. Survey respondents in all samples were predominantly well-educated; more than 94% had completed high school. Less than 23% of respondents in any of the samples were living in poverty or had low incomes. In both surveys, the values for completed education are reasonably similar. Overall, cigarette smoking (defined as the percentage of people who self-reported cigarette use) declined in the entire adult sample by 4.5 percentage points (16% in 2001 and 11.5% in 2004), but in the subsample aged 50 or older by only 2 percentage points (11.4% in 2001 vs 9.4% in 2004).

Two multivariate logistic regression models that controlled for demographic correlates were used to estimate odds of self-reported smoking between the 2 surveys, stratified by the entire sample and the older adult subsample. Controlling for age, sex, marital status, and income, the change in odds of self-reported smoking between the 2 surveys for the entire sample was significant; respondents in 2004 were 20.6% less likely to report smoking than were respondents in 2001. However, no significant difference was found in the odds of self-reported smoking between the 2 surveys for the older adult subsample (controlling for the same covariates). For the entire adult sample, minorities, respondents with lower incomes, and unmarried respondents were significantly more likely to report smoking (age was significant, but the odds ratio was small). Being married, older, or in a higher income category was significantly related to decreased odds of self-reported smoking among the older adult subsample (Table 2).

The percentages of respondents reporting social acceptance of smoking in public between the 2 survey periods were similar in both the all-ages and the older adult groups (Table 3). The estimated odds between the surveys concerning attitude toward public smoking were not significantly different for either the entire sample or the older adult subsample, as estimated by the multivariate logistic regression model controlling for smoking status. Holding survey period constant, among the all-ages sample, smokers were 82.9% more likely than nonsmokers to agree with social acceptability of public smoking. Similarly, older smokers were 82.9% more likely to agree with the social acceptability of public smoking than nonsmokers (Table 4).

Finally, the analyses examining the differences in odds of reporting smoking within the older sample yielded insignificant differences for both those aged 50-59 years and those aged 60 years or older. The number of respondents who reported smoking increased for both groups (2.8% for those aged 50 to 59 years and 1.6% for those aged 60 years or older), but the difference between 2001 and 2004 was insignificant when controlling for demographic covariates. The younger survey respondents (aged 50-59 years in 2001 and 53-62 in 2004) who were married and had higher incomes were less likely to report smoking. Results were similar for survey respondents aged 60 or older in 2001 and 63 or older in 2004; being married and having higher incomes were significantly associated with decreased odds of reporting smoking. Increasing years of age among those aged 60 or older was also significantly associated with a decrease in odds of reporting smoking; however, as was the case earlier, the magnitude of the odds ratio was small (odds ratio [OR], 0.98; 95% confidence interval [CI], 0.94-0.99).

## Discussion

This study demonstrated a significant decrease in overall self-reported smoking reported among all adults in Fort Collins after a public smoking ordinance was implemented. However, this difference was not found in adults aged 50 years or older. This finding suggests that such polices may not have the same effect among older smokers as among younger smokers.

Clean-air ordinances illustrate Rose's paradox of prevention, which posits that population-targeting interventions must have widespread reach to achieve positive impact on overall population health, even though only a portion of the population will actually be directly influenced to change (21,22). In the case of this study, the ordinances were associated with a communitywide reduction in self-reported smoking; however, more detailed analyses indicated that not all groups were influenced equally. We recommend detailed evaluation of the effect of a communitywide intervention and multiple intervention strategies tailored to specific groups not equally affected by the broader intervention to ensure their needs are met equitably.

When both younger (aged 50-59) or older (aged 60 or older) subsamples were examined, we found no significant difference in odds of reporting smoking between the 2 survey periods. Long-term smokers may develop a false sense of security because they have smoked so long with few perceived health consequences and, therefore, see little benefit to cessation (6). Older smokers thus may not be influenced to the same degree as younger smokers by a public smoking ordinance and may require a different approach to encourage cessation, although this may be an artifact of factors unique to this setting. Because adults aged 50 or older may spend less time in places where such an ordinance is in effect, a workplace or public smoking ordinance may not reach them. A possible approach to addressing smoking cessation that focuses on the needs of older adults is individual counseling, supported by pharmacologic intervention (23). This approach is being implemented in Fort Collins, along with group counseling, with promising initial results.

Our logistic regression models suggest that the smoking ordinance had little influence on the overall attitude toward the social acceptability of public smoking, both among all adults and among older adults. This potentially counterintuitive finding may reflect when the samples were drawn in relation to their temporal proximity to the ordinance being implemented. The social acceptability of public smoking may have declined sometime before the actual implementation of the ordinance (perhaps beginning before the first survey in 2001, when more than 50% of people surveyed in 2001 disagreed that public smoking was socially acceptable). This possible decline may have catalyzed community discussion and social engagement and helped to get the ordinance passed.

This study has limitations in both its data collection method and generalizability. The 2001 survey collection occurred close to the implementation of the smoking ordinance, potentially skewing the measurement of attitude toward public smoking; many respondents may have already disagreed with public smoking. Furthermore, the surveys were cross-sectional. The analysis adjusted ages to sample a similar cohort across the 2 survey periods, but the samples are not identical. However, our interest is in looking at overall population cigarette smoking rather than at individual changes in smoking behavior. Ideally, the same respondents would have been surveyed at both survey periods. Finally, the time between implementation of the ordinance and measurement of smoking behavior was short, not allowing the full effect of the ordinance to be measured. Examination of similar variables from future cross-sectional assessments may show additional declines.

To examine the potential effect of the ordinance on the population as a whole, the logistic regression models accounted for variance introduced into the samples through various sociodemographic variables. By controlling for these sociodemographic factors, these models factor out differences between categories of age, sex, racial/ethnic and marital status, and income. The samples were more affluent than the national average and originated from only 1 geographic location, making generalizablity to a broader context difficult.

Another limitation is that the data are self-reported, which potentially introduced response bias into the dataset. Measures of smoking that are self-reported are potentially less accurate than measures obtained using biomarkers. However, self-reported smoking has been correlated with biochemical verification of nicotine use (ie, urinary cotinine) (24). Finally, no information was collected on whether respondents were making individual efforts to limit or stop smoking.

These analyses did not show a significant decrease in the likelihood of reported smoking among older adults, but this finding does not imply that older adults are less likely to stop smoking than younger adults. Several studies have shown that older adults are as likely to be successful at quitting smoking as younger adults, if not more likely; however, interventions targeting older adults may require unique approaches (25-28). The smoking ban did not specifically target environments likely to be frequented by older populations, which may have been a factor in the ordinance's apparent lack of influence in this population.

Future research should focus on further examining changes in cigarette smoking among various subgroups of a population (eg, in categories of age, race/ethnicity, sex, income, and education) and among a broader sample of the national population using the same sample across survey periods. The use of multiple approaches toward smoking cessation, including both broadly focused policy and environmental approaches and interventions specifically tailored for subpopulations not influenced by the broader policy or environmental changes, may expand the effectiveness of interventions that target this public health problem.

## Figures and Tables

**Table 1:** Demographic Characteristics of Respondents, Survey on Current Smoking Status and Attitudes Toward Smoking, by Age, Fort Collins, Colorado, 2001 and 2004

Characteristic	All Adults		Adults Aged ≥50	

	2001 Aged ≥18 (N = 1,680)^a^	2004 Aged ≥21 (N = 1,689)^a^	2001 Aged ≥50 (n = 670)^a^	2004 Aged ≥53 (n = 715)^a^
Age, mean (SD), y	45.8 (17.5)	50.2 (15.9)	63.6 (10.8)	65.6 (9.8)
Female, %	64.0	65.5	64.4	65.4
Married, %	59.0	64.8	64.3	58.4
Non-Hispanic white, %	91.6	93.9	91.6	92.6
High school graduate, %	96.2	96.2	94.6	94.3
Low-income or living in poverty, %^b^	22.3	17.2	16.5	19.7

a The number of survey respondents differs across tables because of small differences in missing data across the different variables used.

b Poverty was defined as household income at or below the federal poverty guidelines for the year of the survey and low-income as having a household income 101% to 185% of the federal poverty guidelines at the time of the survey.

**Table 2:** Odds of Being a Smoker Among Adult Respondents, Survey on Current Smoking Status and Attitudes Toward Smoking, by Age, Fort Collins, Colorado, 2001 and 2004

Variable	All Adults (N = 3,063)^a^		Adults Aged ≥50 y (n = 1,219)^a^	

	OR (95% CI)	*P* Value	OR (95% CI)	*P* Value
**Survey year **				
2004	0.79 (0.64-0.99)	.04	0.91 (0.62-1.37)	.68
2001	1.00 [Reference]		1.00 [Reference]	
**Age^b^**				
>18 y	0.99 (0.98-0.99)	<.001	0.95 (0.93-0.98)	<.001
18 y	1.00 [Reference]		1.00 [Reference]	
**Sex**				
Female	1.06 (0.84-1.34)	.62	0.74 (0.48-1.15)	.18
Male	1.00 [Reference]		1.00 [Reference]	
**Marital status^c^**				
Married	0.43 (0.34-0.54)	<.001	0.30 (0.20-0.46)	<.001
Unmarried	1.00 [Reference]		1.00 [Reference]	
**Race/ethnicity^d^**				
Non-Hispanic white	0.87 (0.77-0.97)	.02	0.64 (0.31-1.32)	.23
Other	1.00 [Reference]		1.00 [Reference]	
**Income^e^**				
Nonpoverty	0.70 (0.59-0.83)	<.001	0.52 (0.38-0.71)	<.001
Poverty and low-income	1.00 [Reference]		1.00 [Reference]	

Abbreviations: OR, odds ratio; CI, confidence interval.

a The number of survey respondents differs across tables because of small differences in missing data across the different variables used.

b 18 y was minimum age in dataset. OR indicates change in odds of smoking for each additional year of age above 18 years.

c Marital status was collected as married, widowed, divorced/separated, single never married, and living with partner, then converted to these dichotomous variables.

d Racial/ethnic status was collected as African American, white non-Hispanic, Hispanic/Latino, Asian/Pacific Islander, and Native American/Alaskan Native, then converted into these dichotomous variables. Hawaiians and Pacific Islanders were excluded from analysis (n = 5 for entire sample).

e Poverty was defined as household income at or below the federal poverty guidelines for the year of the survey and low-income as having a household income 101% to 185% of the federal poverty guidelines at the year of the survey.

**Table 3:** Attitudes Toward Smoking^a^ Among All Adults and Among Those Aged 50 or Older, Survey on Current Smoking Status and Attitudes Toward Smoking, Fort Collins, Colorado, 2001 and 2004

Response	All Adults, %		Adults Aged ≥50, %	

	2001 (N = 1,654)^b^	2004 (n = 1,653)^b^	2001 (N = 538)^b^	2004 (n = 685)^b^
Strongly agree	4.7	4.9	5.4	5.0
Agree	15.1	10.9	9.2	8.0
Neither agree nor disagree	20.6	20.6	18.3	20.2
Disagree	30.9	29.6	31.0	27.7
Strongly disagree	28.8	34.0	36.1	39.1

a Level of agreement with the statement, "It is socially acceptable to smoke in public."

b The number of survey respondents differs across tables because of small differences in missing data across the different variables used.

**Table 4:** Relative Likelihood of Agreeing^a^ That Smoking Is Socially Acceptable, Among All Adults and Those Aged 50 or Older, Survey on Current Smoking Status and Attitudes Toward Smoking, Fort Collins, Colorado, 2001 and 2004

Variable	All Adults (N = 3,252)^b^		Adults Aged ≥50 (n = 1,307)^b^	

	OR (95% CI)	*P* Value	OR (95% CI)	***P* Value**
**Survey year**				
2004	1.08 (0.93-1.25)	.32	0.94 (0.74-1.20)	.66
2001	1.00 [Reference]		1.00 [Reference]	
**Smoking status**				
Smoker	0.17 (0.14-0.21)	<.001	0.17 (0.11-0.26)	<.001
Nonsmoker	1.00 [Reference]		1.00 [Reference]	

Abbreviations: OR, odds ratio; CI, confidence interval.

a Survey respondents who agreed or strongly agreed with the statement, "It is socially acceptable to smoke in public."

b The number of survey respondents differs across tables because of small differences in missing data across the different variables used.
